# Pica in iron deficiency: a case series

**DOI:** 10.1186/1752-1947-4-86

**Published:** 2010-03-12

**Authors:** Yasir Khan, Glenn Tisman

**Affiliations:** 1 Department of Medical Oncology, Whittier Cancer Research Building, Bailey Street, Whittier, California, 90601, USA; 2 St Georges University School of Medicine, Grenada, West Indies

## Abstract

**Introduction:**

Pica is an unusual condition where patients develop cravings for non-nutritive substances that can cause significant health risks. We report three patients with pica, two of them showing evolutionary changes associated with pica and the third demonstrating a peculiar nature of pica, which has yet to be reported.

**Case presentation:**

We describe three patients who presented with symptoms of pica. The first patient is a 36-year-old Caucasian woman who had dysfunctional uterine bleeding associated with daily ingestion of two super-sized cups of ice as iced tea. The second patient is a 62-year-old Caucasian man who presented with bleeding from colonic polyps associated with drinking partially frozen bottled water. Lastly, the third patient, a 37-year-old Hispanic woman, presented with dysfunctional uterine bleeding and habitually chewed rubber bands. All three patients presented with hematological parameters diagnostic for iron deficiency anemia.

**Conclusion:**

Pica has been practiced for centuries without a clear etiology. We have noticed that the younger community of academic and community physicians are not aware of the importance of complaints related to pica. None of our patients we describe here, as well as their primary care physicians, were aware of the importance of their pica related symptoms.

Pica symptoms abated in one of our patients upon iron supplementation, while the other two are currently under treatment as of this writing. We believe pica is an important sign of iron deficiency that should never be ignored, and the craving for any unusual substance should compel clinicians to search for occult blood loss with secondary iron deficiency.

## Introduction

Pica is an unusual craving for and ingestion of either edible or inedible substances. The condition has been described in medical journals for centuries [1-3]. One of the first cases of pica was noted in 6th century AD and was observed in a pregnant woman [1]. Since then, many cases of pica have been reported where patients have acknowledged ingesting ice cubes (pagophagia), clay (geophagia), dried pasta (amylophagia), chalk, starch, paste, Kayexalate resin (resinphagia), tomatoes, lemons, cigarette butts, hair, lead, and laundry starch (for example, Argo out of the box) [1-7]. Although pica is most prominent in individuals with developmental disabilities, it has been observed in men and women of all ages and ethnicity, but is more prevalent among the lower socioeconomic classes [3,4]. Worldwide, 25% to 33% of all pica cases involve small children, 20% are pregnant women, and 10% to 15% are individuals with learning disabilities [8]. A small percentage of patients have iron deficiency anemia.

Pica poses significant health risks that often require medical interventions. These patients are susceptible to electrolyte and metabolic disorders, lead and mercury poisoning, hypokalemia (from resinphagia), parasitic infections, tooth wear, intestinal obstruction, and various problems of the gastrointestinal tract [3,6-9]. The exact etiology of pica remains unclear, but it is significantly associated with iron deficiency anemia [1,2,6,8-10]. When associated with iron deficiency, most physicians believe that pica is an effect rather than a cause [2,10].

Surprisingly, we found that the majority of primary care physicians are unaware of pica symptoms. Over a period of 30 years, our group has evaluated a monthly average of one to two patients with iron deficiency. We have also noted that although the ingestion of excessive amounts of ice (pagophagia) is an unusual symptom, its presence has invariably been associated with documented cases of iron deficiency anemia.

Here we present three patients who demonstrated subtle changes in pica associated with iron deficiency. We believe that this was due to advances in technology and changing cultural customs. Each patient fully meets the criteria for pica from Diagnostic and Statistical Manual of Mental Disorders [5] and suffered from severe iron deficiency anemia.

## Case presentation

Our first patient is a 36-year-old Caucasian woman with dysfunctional uterine bleeding. She has experienced unusually heavy periods of bleeding for 12 months prior to her presentation to our medical facility. Her heavy bleeding required approximately two boxes of tampons for every month of her menstrual cycle. Her hemoglobin level was 10.2 g/dl, mean corpuscular volume (MCV) was 68 fl and serum ferritin was 8 ng/ml. Her peripheral blood smear revealed poikilo and microcytosis. When asked about ice-cube eating, she stated that she drank and sucked ice cubes from at least two super-sized McDonald's cups filled with ice on a daily basis. The ice was part of a super-sized tea.

Our second patient is a 62-year-old Caucasian man who presented with bleeding from colonic polyps. His hemoglobin level was 8 g/dl and his serum ferritin was 11 ng/ml. His Wright-Giemsa-stained peripheral blood smears revealed anisocytosis with microcytosis, which was characteristic of iron deficiency. He claimed to drinking extremely cold water. He would place his bottled water in the freezer so that it would form an adherent patch of ice that encompassed the container, thus reducing its temperature. He would drink his iced water from three to four of these containers daily, but he denied eating ice cubes. He underwent colonoscopic polypectomy and iron replacement therapy. His craving for cold bottled water abated within two months of therapy.

Our third patient is a 37-year-old Hispanic woman with dysfunctional uterine bleeding for approximately one year. Her hemoglobin level was 7 g/dl, MCV was 64 fl and serum ferritin was 4 ng/ml. Her peripheral blood smear revealed anisocytosis and poikilocytosis with microcytosis. This patient had been chewing rubber bands for at least six months prior to dilation and curettage. When asked to be specific, she stated that she would chew three or four thin, preferably cream-colored rubber bands continuously throughout the day. We were surprised to see that she brought her favorite rubber bands to the hospital so she could resume chewing after she had awakened from anesthesia.

## Discussion

Although observed since antiquity, pica remains a mysterious and fascinating occurrence. It seems to be strongly associated with iron deficiency anemia, and in the majority of cases the unusual eating and chewing behavior disappears upon iron supplementation [1,6,10,11]. Several hypotheses exist about why iron deficiency causes pica, including physiological mechanisms; however, there is no single agreed upon explanation [1,4]. Pica has been linked to factors of age, gender, religion, culture, nutritional deficiency, stress, and mental development [4]. When associated with iron deficiency, it is believed to be a symptom of the deficiency rather than its cause [2,10]. Occasionally, pica practices cause other nutritional deficiencies such as hypokalemia (clay and Kayexalate ingestion [8,12]).

We present these cases because they represent subtle changes in the classical symptom complexes of pica. This may probably be the result of advances in technology and changes in culture. When initially described, pagophagia was defined as the excessive ingestion of ice cubes from ice trays and the ingestion of ice scraped from the wall of the freezer [1]. With the advent of ice cube makers and auto defrosters, the presentation of pagophagia has changed in a subtle manner as described in two of our patients. Now we observe a subtler ingestion and/or sucking of ice cubes from large super-sized McDonald's-like cups and from the use of popular bottled water containers that have been frozen. Moreover, the third patient we describe is the first report of rubber band chewing as a manifestation of iron deficiency anemia. Recently, Hackworth and Williams presented three cases where patients with sickle cell anemia readily ingested foam rubber, and Kushner *et al. *presented two cases where patients developed pagophagia after gastric bypass surgery [2,5].

Since iron deficiency may cause glossal pain, it has been proposed that patients with anemia choose to chew ice for its analgesic properties; however, rubber bands and foam do not have any known analgesic properties [10]. There have been several theories explaining the causes of pica. Earlier investigators proposed that pica practices compensated for nutritional deficiencies, such as iron or zinc, but this idea was discarded as ice, rubber, foam and several other items, consumed by those who practice pica, do not have any known nutritional value. Other theories suggest possible psychosocial problems, family stress, obsessive-compulsive disorders, or merely the enjoyment of taste and texture (the crunch of Argo starch out of the box or of clay cookies) of the item being consumed [12].

Interestingly, pica is practiced when a patient is least supervised. Patients are also secretive of their pica habits and are often reluctant to mention it. Pica symptoms will thus go unnoticed unless the physician specifically addresses them [2,13,14]. All three of our patients, as well as their primary care physicians and academic supervisors, were unaware of the symptoms of pica and what these represented.

Earlier diagnosis of pica can prove beneficial especially in the presence of an occult, bleeding malignancy or if the patient is pregnant [2]. Pica in pregnancy is not uncommon and, if unnoticed, may put both the mother and fetus at risk [3,15].

## Conclusion

We hope that this report reminds physicians of the importance and diagnostic utility of pica symptoms associated with iron deficiency, as well as the evolution of its symptoms as a result of changing technology and culture. In the presence of pica, the physician is obligated to evaluate the patient for occult blood loss and iron deficiency.

## Competing interests

The authors declare that they have no competing interests.

## Authors' contributions

GT is the consulting hematologist of our patients reported, was responsible for the general scope and ideas for the research, and contributed in writing the manuscript. YK conducted the literature research study and wrote the manuscript under the guidance of GT. Both authors read and approved the final manuscript.

## Consent

Written informed consent was obtained from our patients for publication of this case report and any of the accompanying images. A copy of the written consent is available for review by the Editor-in-Chief of this journal.
